# A multi-sensor approach to improve interpretability of the 6-min walk test as an outcome in muscular dystrophies: an observational study

**DOI:** 10.1093/braincomms/fcaf205

**Published:** 2025-06-05

**Authors:** Aisha Sheikh, Mireia Claramunt-Molet, Karen Rudolf, Carolina Migliorelli, Sebastian Idelsohn-Zielonka, Felip Miralles, John Vissing

**Affiliations:** Copenhagen Neuromuscular Center, Department of Neurology, Rigshospitalet, University of Copenhagen, Copenhagen 2100, Denmark; Unit of Digital Health, Eurecat, Centre Tecnològic de Catalunya, Barcelona 08005, Spain; Ephion Health, Barcelona 08005, Spain; Copenhagen Neuromuscular Center, Department of Neurology, Rigshospitalet, University of Copenhagen, Copenhagen 2100, Denmark; Unit of Digital Health, Eurecat, Centre Tecnològic de Catalunya, Barcelona 08005, Spain; Unit of Digital Health, Eurecat, Centre Tecnològic de Catalunya, Barcelona 08005, Spain; Ephion Health, Barcelona 08005, Spain; Unit of Digital Health, Eurecat, Centre Tecnològic de Catalunya, Barcelona 08005, Spain; Ephion Health, Barcelona 08005, Spain; Copenhagen Neuromuscular Center, Department of Neurology, Rigshospitalet, University of Copenhagen, Copenhagen 2100, Denmark

**Keywords:** muscular dystrophies, endpoint, biomechanics, 6-min walk test

## Abstract

The 6-min walk test (6MWT) is commonly used to assess physical function and endurance. The purpose of this observational study was to test a newly developed biomechanical system, Ephion Mobility system, and establish whether these biomechanical measures can improve the objectivity and interpretability of the 6MWT as an outcome measure. Fifty-eight participants took part in this study: 20 participants with myotonic muscular dystrophy (DM1), 18 with Becker muscular dystrophy (BMD) and 20 healthy controls for comparison. Every DM1 participant was classified using Muscular Impairment Rating Scale, and every BMD participant was classified using the Vignos scale. Each participant performed the 6MWT while wearing the Ephion Mobility system sensors, which recorded walking distance, surface electromyography (sEMG), heart rate (HR), ground reaction force (GRF), trunk vertical acceleration and joint kinematics. Both patient groups exhibited significantly shorter walking distance, lower cadence and shorter step length. Lower HR was found in both patient groups; this increased as disease severity increased. Limited range of motion was observed during the entire walk test in both patient groups. The DM1 group showed gait alterations during toe-off with reduction in hip and knee extension and a delayed and reduced plantar flexion. As disease severity increased, visual interpretation of sEMG in the DM1 group exhibited lower amplitude in the proximal muscles. The BMD group exhibited gait alterations during contact, stance and toe-off phases. Flat foot landing was observed in the BMD group along with reduced hip flexion and ankle dorsiflexion. During stance, they were unable to extend the hip and flex the knee and muscle activity increased, and reduced plantarflexion was observed during toe-off. The GRF was lower during heel strike and higher during mid-stance, and trunk vertical acceleration was close to zero during the contact phase in both patient groups. A visual interpretation of the gait patterns showed differences among disease severity levels. Our findings show that the Ephion Mobility system can determine biomechanics during walking and is coupled with distinct patterns of walking in BMD and DM1. Ephion Mobility system is useful in obtaining observational differences among disease severities and may be responsive to progression or treatment-induced improvement. The system adds value to improving interpretability of the 6MWT and additionally be used in assessing other functional capabilities, such as sit-to-stand and stair climbing.

## Teaser

The study evaluates Ephion Mobility system’s ability to enhance the 6-min walk test by identifying biomechanical patterns in Becker muscular dystrophy and myotonic dystrophy type 1. The portable, non-invasive system improves the 6-min walk test and provides qualitative differences in disease severity, aiding clinical assessments, therapeutic monitoring and tracking disease progression.

## Graphical Abstract

**Graphical Abstract:**
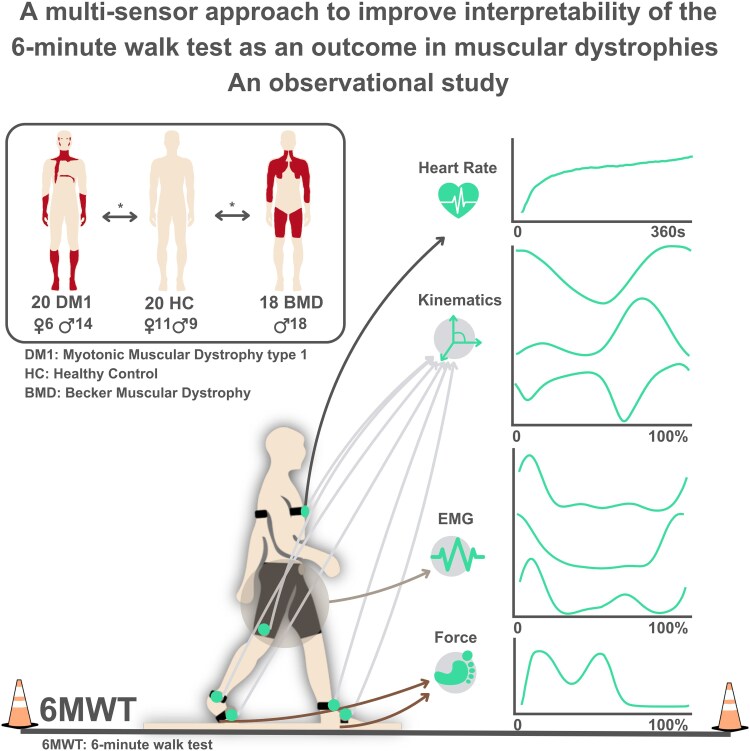

## Introduction

The 6-min walk test (6MWT) is commonly used to assess physical function and capacity in various patient populations and as an endpoint in intervention studies and clinical trials. Official guidelines for the 6MWT were presented in 2002 by the American Thoracic Society.^1^ The 6MWT evaluates the response of all systems involved in walking. The test has been applied widely to assess functional capacity in neuromuscular disorders and has proved to be a reliable outcome measure in myotonic dystrophy,^2^ spinal and bulbar muscular atrophy,^3^ chronic spinal poliomyelitis,^4^ Pompe disease,^5^ Duchenne muscular dystrophy (DMD)^6^ and chronic inflammatory polyneuropathy.^7^

The 6MWT is submaximal self-paced test and involves measuring the distance a patient can walk on a level course in 6 min. The test is simple to execute and does not require sophisticated expensive equipment. Moreover, the 6MWT reflects activities of daily living.

In myotonic muscular dystrophy type 1 (DM1) and Becker muscular dystrophy (BMD), the test has been used as an outcome measure, in conjunction with other tests, to assess changes in physical function.^8-12^ The test assesses walking distance and endurance, but the test is subject to great variability based on patient motivation, day-to-day variation and learning effects.^13-15^ In this study, we used the 6MWT while assessing gait biomechanics using the Ephion Mobility system. Coupling the 6MWT with the analysis of gait biomechanics and heart rate (HR) at the same time can help understand exertional efforts and gait dynamics besides the crude distance of the test and how these parameters differ among disease groups, thus potentially improving the interpretability of the 6MWT. Therefore, the purpose of this observational study was to establish a more objective and clinically relevant assessment of the 6MWT by adding measures of surface electromyography (sEMG) activity, joint kinematics and weight distribution during gait by using and easy-to-use motion analysis system.

## Materials and methods

### System characteristics

**Figure 1:**
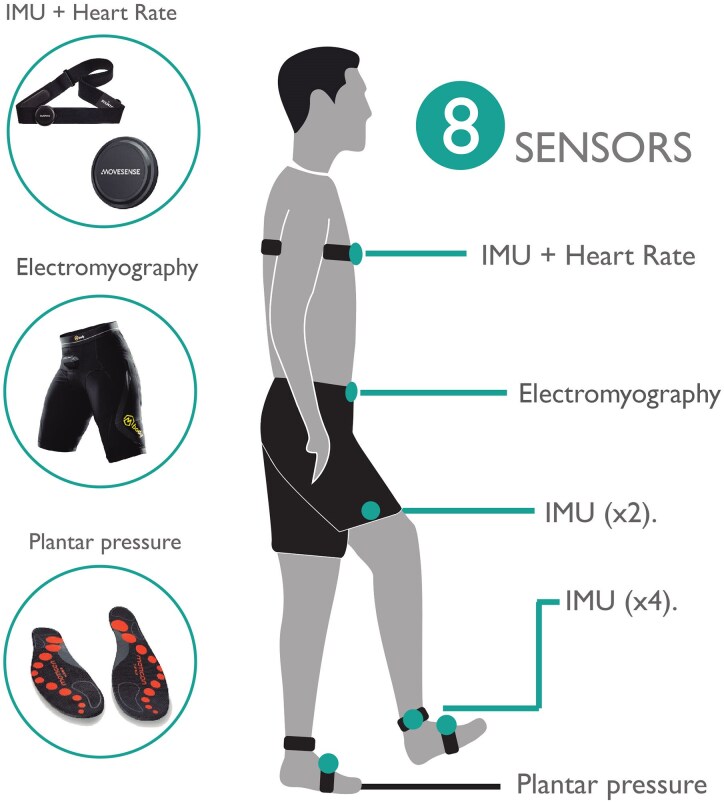
**Sensor placement.** The figure illustrated the placement of the eight sensors, including the IMU, the shorts with integrated surface electromyography and the plantar pressure insoles.

The Ephion Mobility system was used to measure gait biomechanics during the 6MWT. This system has been utilized in a previous study.^16^ The system collects synchronized data from eight commercially available wearable sensors attached to different parts of the body (Fig. 1).

HR in beats per minute (bpm) was measured using a strap, which was applied below the chest.^17^

Inertial measurement unit (IMU) sensors^17^ were placed below the sternum; this sensor simultaneously captured HR data, lateral side of the thigh and shank, and integrated inside the plantar pressure insoles for measuring joint kinematics. Anatomical landmarks for the sensors were chosen based on avoiding areas close to joints and soft tissue areas (Fig. 1) and considering that the whole-body segment moves in the same way.^18-22^ All the participants followed the same protocol, and two physical therapists were the ones in charge of placing and reviewing the positioning of the sensors.

IMU sensors measured accelerations due to gravity, in g (9.8 m/s2), and angular velocities, in degrees per second (°/s), about the *x*-, *y*- and *z*-axes of different body segments (trunk, thigh, shank and foot).

sEMG sensors (dry electrodes made of silver-coated textile yarn) integrated in custom-made, size-appropriate shorts were used to record muscle activity of the hamstrings, gluteus maximus and quadriceps muscles.^23^ For this, we followed the recommendations stated by Finni *et al*.^24^ in their validation study. The best-fitting shorts were chosen for each participant, ensuring the embedded electrode had direct contact with the surface of the skin and a small amount of water was applied to the electrodes to ensure adequate signal conduction. sEMG signals were captured at 1000 Hz; expressed in µV, average rectified data were transmitted wirelessly at 25 Hz to a cellphone. Signal-processing methods and validation have been described previously.^25,26^ The purpose of this study with respect to sEMG was not to measure single muscle activity but a group of muscles. The reliability of the shorts, which facilitated the positioning of the sensors in a large area, was tested and validated in previous studies.^27^  ^,28^

Pressure sensors measuring ground reaction force (GRF), measured in Newton (N), were integrated in insoles, which were size-appropriate and used to obtain information on the body’s weight distribution during gait.^29^

### Ethics

The study was conducted at the Copenhagen Neuromuscular Center at the National University Hospital, Rigshospitalet in Copenhagen, Denmark, from March 2020 to July 2021 in accordance with the ethical standards laid down in the 1964 Declaration of Helsinki and its later amendments and was approved by the Danish National Committee on Health Research Ethics (H-19027533) and the Danish National Data Safety Committee (J.nr. P-2019-267). Before data collection, written informed consent was obtained from all participants. Written informed consent was also obtained from the participants for the publication of any potentially identifiable images or data included in this article.

### Participants

Inclusion criteria were (i) 18 years of age or older, (ii) genetically verified DM1 or BMD, (iii) ability to walk without assistive device and (iv) able to comprehend and adhere to participation requirements. We wished to investigate the feasibility and applicability of capturing additional data during the 6MWT when walking was compromised by either proximal or distal weakness. Hence, we chose to include participants with BMD with predominantly proximal involvement and participants with DM1 with predominantly distal involvement. Patients diagnosed with BMD and DM1 from our outpatient clinic at Copenhagen Neuromuscular Center who fulfilled the inclusion criteria were offered participation in the study. Each participant with BMD was also assessed for disease severity using the Vignos lower extremity scale. The Vignos lower extremity scale is used to describe lower extremity functional ability in boys with DMD and has also been used in patients with BMD.^30^ Furthermore due to a high prevalence of heart involvement in this patient population, we screened each patient’s medical record for any pre-existing heart transplant, pacemaker or need for heart medication. The DM1 participants were assessed for disease severity using the Muscular Impairment Rating Scale (MIRS), a reliable scale to assess muscle impairment in DM1.^31^ For this observational study, where differences in the biomechanics among the participants are unknown, a power calculation was not feasible. The cohorts were predetermined to be composed of 20 participants in each group, based on availability of patients and to capture a wider disease spectrum in the two patient groups and a healthy control (HC) group was recruited for comparison. The participants in the control group were required to have no lower limb issues that would affect their walking capabilities.

### Study design

Participation consisted of two visits to perform the 6MWT. Before walking, participants were equipped with the eight sensors attached to different parts of the body and an Ephion Mobility system smart device was used to collect the data. Two physical therapists were responsible for placement and verification of each sensor during both visits.

The 6MWT was carried out on a 25-m levelled, undisturbed and well-lit hallway. Two traffic cones defined the start and end of the walkway. A chair was available in case the participant needed to sit down and rest. Standardized verbal instruction and encouragement were provided to each participant before and during the test.

For safety and connectivity of the sensors, a physical therapist walked behind the participant without talking to or pacing the participant. Number of laps, rest periods and falls were recorded during the test. Borg Scale was used pre- and post-test to determine perceived level of exertion.^32^

Once the 6MWT was complete, the distance walked was instantly available on the smart device and the data recorded from the sensors were sent to a cloud where a comprehensive report of the data could be viewed and used for further analysis.

### Data collection and processing

Recorded data from both visits were calibrated and normalized as follows:

- HR data were obtained directly from the HR sensor during the entire 6MWT, at 1 Hz.
- GRF was normalized to body weight (N/kg), by dividing the 6MWT values by the weight of the subject. The insoles were zeroed by subtracting the residual values recorded during the calibration period, which consisted of lifting each foot for 3 s, from the values recorded during the 6MWT.
- Averaged and rectified sEMG, expressed in µV, of quadriceps, hamstrings and gluteus maximus muscles was normalized to the maximum value recorded during the 6MWT, and the amplitude of the sEMG signal was composed of the collective action potentials from motor units near the electrode. Therefore, sEMG signal of each muscle was divided by its respective maximum value, resulting in unitless measurements of the activation ratio.
- Co-activation indices referring to the timing of simultaneous activation of two muscle groups were calculated by obtaining the common area between the two muscle profiles and dividing by the number of points of the curve.^33^ Co-activation indices were obtained for each pair of muscles: gluteus maximus and quadriceps, hamstring and quadriceps and gluteus maximus and hamstrings.
- Spatial-temporal parameters for every 6MWT were calculated from inertial sensors and GRF data, as described below:
  - The total number of steps taken during the test was determined by identifying cycles in the GRF signal.
  - Cadence, measured in steps per minute (steps/min), was calculated by dividing the total number of steps by the test duration (6 min), presented as mean value.
  - Stride duration, in seconds (s), was calculated by dividing the test duration by half the number of steps since one stride consists of two steps, presented as mean value.
  - The total number of laps completed during the test was detected using data from the trunk’s gyroscope. The number of laps detected was consistent with the number of laps manually recorded. The total distance walked, in metres (m), was determined by multiplying the number of laps by the corridor’s length and adding metres covered after the last lap manually in Ephion Mobility app.
  - Velocity, in metres per second (m/s), was calculated dividing the total distance covered by the test duration in seconds, presented as mean value.
  - Stride length, in metres (m), was calculated by dividing the test distance by half of the total number of steps, presented as mean value.
  - Stride height, in metres (m), was computed per each stride by integrating twice the data from the accelerometer on the insole. This value was then averaged across all strides,^34^ presented as mean value.
  - Stride velocity, in metres per second (m/s), was computed by dividing the stride length by the time taken to complete one stride, presented as mean value.
- Anatomical joint angles in the sagittal plane (flexion and extension) of the trunk, hip, knee and ankle were calculated by combining gyroscope data from the two body segments linked to the joints. First, absolute segment angles were obtained by integrating angular velocity^35^ and correcting the drift.^36^ Then, per each joint, motion absolute segment angle was subtracted from reference absolute segment angle. For example, thigh minus shank to compute knee angle. Trunk segment was used as the reference segment.

Additionally, GRF data were used to identify gait cycles for each leg throughout the 6MWT. Subsequently, sEMG, GRF and kinematics data collected during the 6MWT were segmented based on these gait cycles and then averaged to derive the mean gait pattern. For spatial-temporal parameters, the mean values across all tests were calculated.

### Statistical analysis

Data from the two patient groups, DM1 and BMD, were compared to the results of the HC group. The DM1 group and BMD group were not tested against each other.

Statistical analysis of the spatial-temporal, kinematics, HR, GRF and EMG variables was performed using Mann–Whitney U-test for independent samples and corrected by multiple comparisons using the Bonferroni method. The Mann–Whitney U-test was used to evaluate the null hypothesis that all the samples from controls and patients came from the same population. Mean group differences in the biomechanical patterns (kinematics, EMG and GRF) were assessed using permutation test of independent samples. Additionally, Cohen’s *d* effect size metric was performed between patterns. This analysis was restricted to points showing statistically significant differences between groups (HC, DM1 and BMD) and a threshold of 0.5 was set, showing only medium to large effect sizes. The level of significance for all analyses was set at *P* < 0.05.

To ensure robust comparisons and increase statistical power, we followed the recommendations presented by A’Hern.^37^ We therefore included data from both tests per participant [test and retest (ICC > 0.8)] and data from both sides [right and left (*r*-value > 0.85)].

Due to sample size limitations, statistical analysis was not performed for severity groups; instead, we performed a qualitative observational analysis of the severity groups.

The results of DM1 and BMD were performed per patient group and per subgroups within the two diagnoses according to their disease severity. An additional subgrouping of BMD participants was performed according to presence of heart involvement, such as use of heart medication, heart transplant or pacemaker or no heart involvement.

## Results

A total of 58 participants took part in this study, 20 with DM1 of whom 6 were women, 18 men with BMD of whom 10 had a heart involvement (pacemaker *N* = 1, heart transplant *N* = 1, medication for heart failure *N* = 8) and 8 with no heart involvement and 20 HC of whom 11 were women (Table 1). Despite the age difference being 3–11 years on average among the three groups (Table 1), this age variability does not affect the biomechanics or walked distance in adults according to previous studies.^38-41^

**Table 1:** Demographics of participants with DM1, BMD and HC

	*N*	AGE	HEIGHT (CM)	WEIGHT (KG)	*N* (FEMALE)	*N* (MALE)
**DM1**	20	43.5 ± 10.0	176.1 ± 9.1	75.9 ± 13.8	6	14
**BMD**	18	35.8 ± 10.5	178.2 ± 7	81.7 ± 10.6	N/A	18
**HC**	20	32.8 ± 10.2	175 ± 10.5	72.4 ± 12.4	11	9

DM1, myotonic muscular dystrophy type 1; BMD, Becker muscular dystrophy; HC, healthy control.

### Walking distance

**Figure 2:**
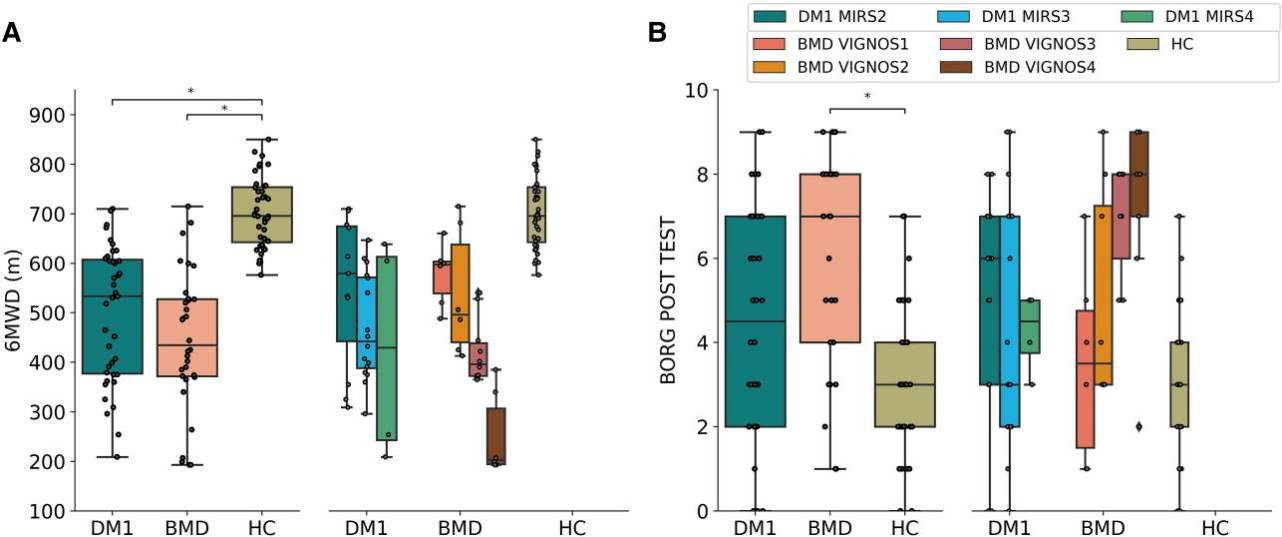
**6MWT walking distance and Borg scale.** (**A**) Boxplots display distance during the 6MWT for DM1, BMD and HC and distance walked according to severity in DM1 (7 patients with severity 2, 11 patients with severity 3, 2 patients with severity 4) and in BMD (*N* = 4 severity 1, *N* = 4 severity 2, *N* = 6 severity 3 and *N* = 4 severity 4). (**B**) Boxplots display Borg scale values post-test in DM1, BMD and HC and Borg scale values according to disease severity in DM1 (7 patients with severity 2, 11 patients with severity 3, 2 patients with severity 4) and in BMD (4 patients with severity 1, 4 patients with severity 2, 6 patients with severity 3 and 4 patients with severity 4). Mann–Whitney U-test, corrected by multiple comparisons using the Bonferroni method, was utilized for the analysis, comparing HC versus DM1 and HC versus BMD. Asterisk (*) indicates a significant difference (*P*_Bonferroni_ < 0.05). DM1, myotonic muscular dystrophy type 1; BMD, Becker muscular dystrophy; HC, healthy control; 6MWD, 6-min walk distance.

There was a significant difference in walking distance between patient groups and HC (*P* < 0.05) (Fig. 2A and Table 2). Mean distance walked during the 6MWT for HC was 703.12 m (±70.35). The perceived level of exertion post-test was significantly different between BMD and HC, but not between DM1 and HC (Fig. 2B). There was a successive decrease in walked distance with increasing disease severity in both patient groups, most marked in participants with BMD (Fig. 2A and Table 2). The decrease in walked distance was paralleled by successive increase in stride duration and drop in velocity and stride length and height (Table 2).

**Table 2:** Comparison of BMD and DM1 6MWT spatiotemporal parameters to health controls

GROUP/PARAMETER (UNIT)	HC	DM1	DM1 (MIRS SCORE)			BMD	BMD (VIGNOS SCORE)			
2MWD		ALL	2	3	4	ALL	1	2	3	4
2MWD (M)	233.61 (27.0)	170.94 (48.71)^a^	188.26 (45.43)	166.3 (39.76)	136.95 (87.81)	153.13 (45.85)^a^	189.96 (25.89)	180.3 (36.03)	148.22 (30.71)	88.52 (23.75
6MWD (M)	703.12 (70.35	501.55 (135.37)	555.71 (137.43	481.2 (108.32	426.75 (226.63	444.62 (140.59)	578.17 (63.02)	534.88 (109.8)	427.18 (65.14)	252.83 (86.28
Cadence (steps/min)	139.3 (8.0)	122.09 (11.09)^a^	127.6 (10.2)	119.7 (9.4)	116.2 (10.3)	107.31 (19.67)^a^	125.7 (6.6)	122.7 (17.1)	101.6 (7.7)	82.3 (11.3)
Velocity (m/s)	2.11 (0.3)	1.6 (0.64)^a^	1.64 (0.39)	1.61 (0.78)	1.5 (0.83)	1.65 (0.87)^a^	1.8 (0.18)	1.87 (0.8)	1.76 (1.18)	0.98 (0.54)
Stride duration (s)	0.87 (0.05)	1.0 (0.09)^a^	0.95 (0.08)	1.01 (0.08)	1.04 (0.09)	1.17 (0.24)^a^	0.96 (0.05)	1.0 (0.13)	1.19 (0.09)	1.52 (0.24)
Stride length (m)	1.52 (0.1)	1.2 (0.32)^a^	1.34 (0.25)	1.17 (0.28)	1.12 (0.45)	1.16 (0.33)^a^	1.37 (0.16)	1.24 (0.16)	1.25 (0.28)	0.72 (0.19)
Stride height (m)	0.22 (0.04)	0.16 (0.05)^a^	0.17 (0.05)	0.16 (0.04)	0.15 (0.07)	0.18 (0.05)^a^	0.2 (0.04)	0.2 (0.05)	0.17 (0.03)	0.14 (0.06)
STRIDE VELOCITY (m/s)	1.77 (0.17)	1.25 (0.38)^a^	1.44 (0.34)	1.18 (0.32)	1.12 (0.53)	1.07 (0.42)^a^	1.44 (0.23)	1.29 (0.33)	1.05 (0.24)	0.5 (0.2)

Table displays walked distance, cadence, velocity, stride duration, length, height and velocity in HC, DM1 and BMD indicated in mean (±SD). Mann–Whitney U-test, corrected by multiple comparisons using the Bonferroni method, was used for the statistical analysis.

^a^Significant difference (*P*_Bonferroni_ < 0.05) between DM1 and HC and between BMD and HC. Values according to disease severity in DM1 and BMD are indicated next to the global pathology group in mean (±SD).

HC, healthy control; DM1, myotonic muscular dystrophy type 1; BMD, Becker muscular dystrophy; m, metre; s, seconds; 2MWD, 2-min walk distance; 6MWD, 6-min walk distance.

### Surface electromyography in myotonic muscular dystrophy type 1 versus healthy control

**Figure 3:**
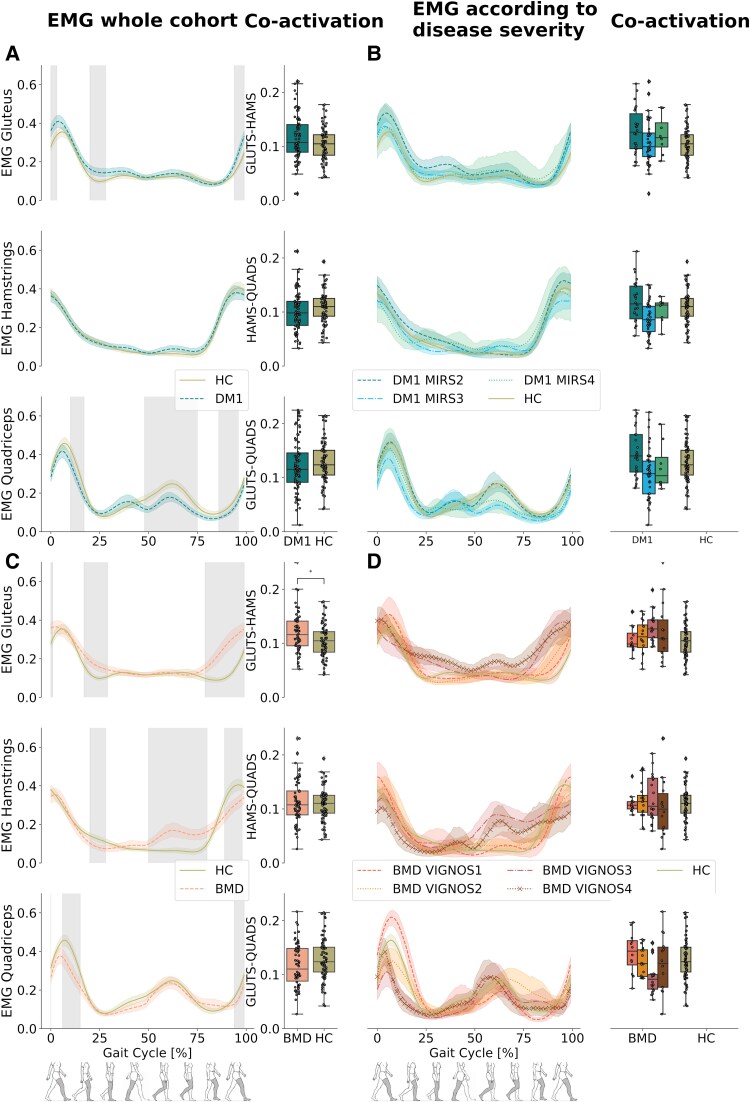
**Electromyography of the gluteus maximus, hamstrings and quadriceps.** (**A**) sEMG amplitude of the gluteus maximus, hamstring and quadriceps muscles in DM1. Grey shaded areas indicate significant difference between DM1 (blue striped line) and HC (solid olive line) during the walk test (*P* < 0.05) along with boxplots displaying muscle co-activation in DM1 and HC. (**B**) sEMG amplitude according to disease severity in DM1 (7 patients with severity 2, 11 patients with severity 3, 2 patients with severity 4) along with boxplots displaying muscle co-activation in DM1 and HC. (**C**) sEMG amplitude of the gluteus maximus, hamstring and quadriceps muscles in BMD. Grey shaded areas indicate significant difference between BMD (orange striped line) and HC (solid olive line) during the walk test (*P* < 0.05) with boxplots displaying muscle co-activation in BMD and HC. (**D**) sEMG amplitude according to disease severity in BMD (four patients with severity 1, four patients with severity 2, six patients with severity 3 and four patients with severity 4) along with boxplots displaying muscle co-activation in BMD and HC. Permutation and D Cohen effect size was used to analyse the grey shaded area, and Mann–Whitney U-test, corrected by multiple comparisons using the Bonferroni method, was used for the analysis for boxplots, comparing HC versus DM1 and HC versus BMD. Asterisk (*) indicates a significant difference (*P*_Bonferroni_ < 0.05). DM1, myotonic muscular dystrophy type 1; BMD, Becker muscular dystrophy; HC, healthy control; GLUTS, gluteus maximus; HAMS, hamstrings; QUADS, quadriceps.

DM1 participants showed significantly lower amplitude in the quadriceps during heel strike and initial swing in comparison with HC and exhibited a higher amplitude in gluteus maximus muscle during heel strike, mid-stance and terminal swing (*P* < 0.05) (Fig. 3A). No significant differences were observed in hamstrings. sEMG co-activation of gluteus maximus and hamstrings was significantly higher in DM1 than in HC (*P* < 0.05) (Fig. 3A). A visual interpretation of the disease severity subgroups showed that the intergroup differences became progressively higher with increasing disease severity (Fig. 3B). No significant differences were found for agonist–antagonist muscles.

### Surface electromyography in Becker muscular dystrophy versus healthy control

Participants with BMD exhibited significantly higher amplitude in the gluteus maximus during mid-stance and terminal swing in comparison with HC (*P* < 0.05). Significant amplitude differences were also found in the hamstrings in BMD group, where we observed a lower amplitude during mid-stance, a higher amplitude between terminal stance and mid swing phase and a lower amplitude during terminal swing phase in comparison with HC (*P* < 0.05). The quadriceps muscles showed significantly lower amplitude during heel strike and mid-stance in comparison with HC (*P* < 0.05) (Fig. 3C). A visual interpretation of the disease severity subgroups showed that the intergroup differences became progressively higher with increasing disease severity (Fig. 3D).

sEMG co-activation of gluteus maximus and hamstrings was significantly higher in BMD than in HC (*P* < 0.05). Co-activation increased with increasing disease severity. No significant differences were found for agonist–antagonist muscles.

### Heart rate

**Figure 4:**
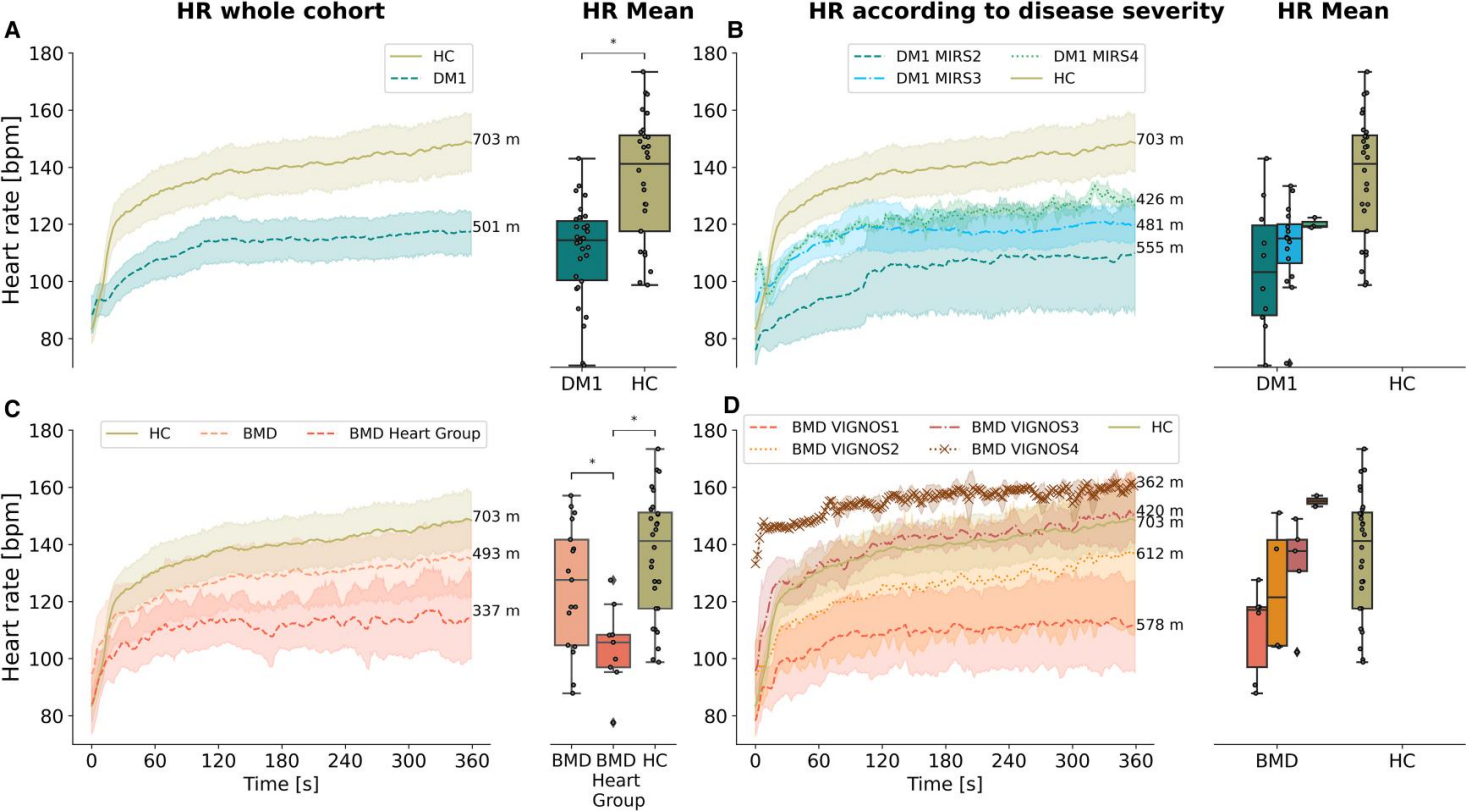
**HR.** (**A**) HR values of HC (olive solid line) and DM1 (blue striped line) along with boxplots of mean HR values in DM1 and HC. (**B**) HR values according to disease severity in DM1 (7 patients with severity 2, 11 patients with severity 3, 2 patients with severity 4) and HC along with boxplots of mean HR values in DM1 and HC. (**C**) HR values of BMD with heart involvement (*N* = 10) (orange striped line) and BMD without heart involvement (*N* = 8) (light orange dotted line) along with boxplots of mean HR values in BMD and HC. (**D**) HR values in BMD according to disease severity (four patients with severity 1, four patients with severity 2, six patients with severity 3 and four patients with severity 4) and HC along with boxplots with mean HR values in BMD and HC. Mann–Whitney U-test, corrected by multiple comparisons using the Bonferroni method, was utilized for the analysis, comparing HC versus DM1 and HC versus BMD. Asterisk (*) indicates a significant difference (*P*_Bonferroni_ < 0.05). HR, heart rate; BPM, beats per minute; DM1, myotonic muscular dystrophy type 1; BMD, Becker muscular dystrophy; HC, healthy control.

During the 6MWT, DM1 group had a significantly lower mean HR compared to HC (*P* < 0.05) (Fig. 4A). Among BMD patients without heart involvement, the mean HR was no different from the group. However, in the subgroup with heart involvement (*N* = 10), the mean HR was significantly lower than the group without heart involvement (*N* = 8) and the HC group (*P* < 0.05) (Fig. 4C). Additionally, visual observations of the results indicated differences in severity groups. As disease severity increased, both patient groups exhibited higher HRs during the walk test, indicating that more severely affected patients exerted a greater effort (Fig. 4B and D). Reflective of this greater effort, BMD patients with higher disease severity also had higher Borg scale scores at the end of the 6MWT (Fig. 2B).

### Ground reaction force and trunk vertical acceleration

**Figure 5:**
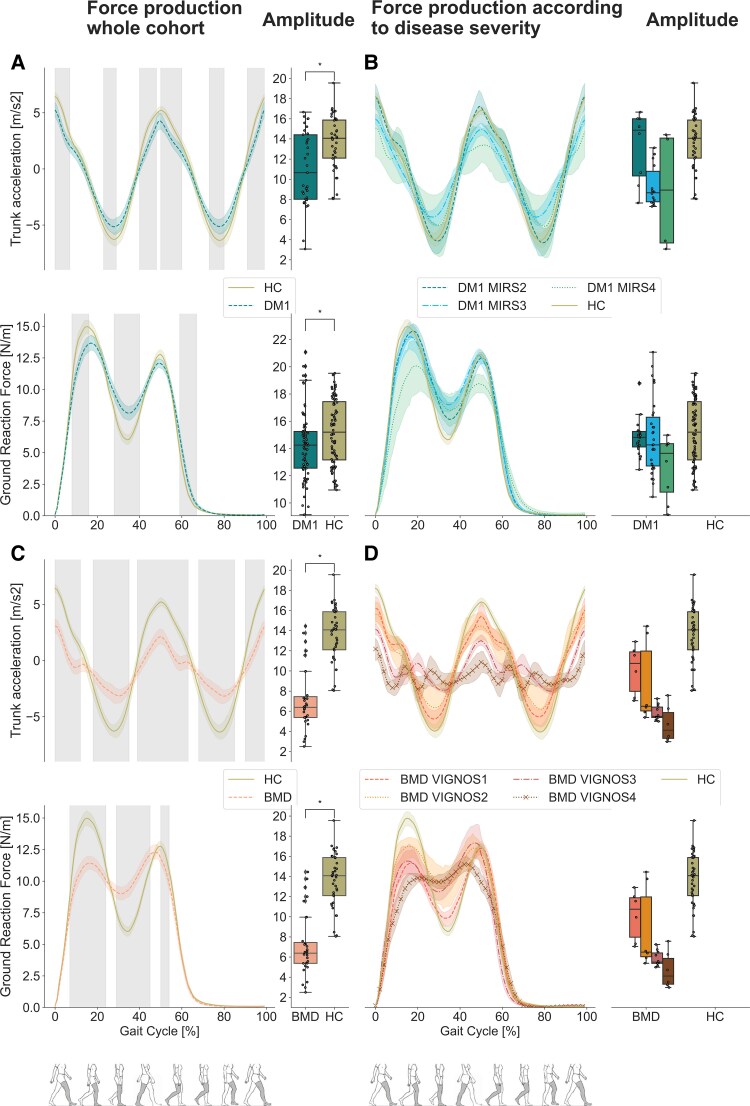
**Trunk acceleration and GRF.** (**A**) Trunk acceleration (m/s^2^) (top) and GRF (Newton/kg) (bottom) in DM1 (blue striped line) where the grey shaded area indicates a significant difference in comparison with HC (olive solid line) during the walk test (*P* < 0.05) along with boxplots for trunk acceleration (top) and GRF (bottom) in DM1 and HC. (**B**) Trunk acceleration (top) and GRF (bottom) according to disease severity in DM1 (7 patients with severity 2, 11 patients with severity 3, 2 patients with severity 4) along with boxplots for trunk acceleration (top) and GRF (bottom) in DM1 and HC. (**C**) Trunk acceleration (top) and GRF (bottom) in BMD (orange striped line) where the grey shaded area indicates a significant difference in comparison with HC (olive solid line) during the test (*P* < 0.05) along with boxplots for trunk acceleration (top) and GRF (bottom) in BMD and HC. (**D**) Trunk acceleration (top) and GRF (bottom) according to disease severity in BMD (four patients with severity 1, four patients with severity 2, six patients with severity 3 and four patients with severity 4) along with boxplots for trunk acceleration (top) and GRF (bottom) in BMD and HC. Permutation and D Cohen effect size was used to analyse the grey shaded area, and Mann–Whitney U-test, corrected by multiple comparisons using the Bonferroni method, was used for the analysis for boxplots, comparing HC versus DM1 and HC versus BMD. Asterisk (*) indicates a significant difference (*P*_Bonferroni_ < 0.05). N, Newton; m, meter; s, seconds; DM1, myotonic muscular dystrophy type 1; BMD, Becker muscular dystrophy; HC, healthy control.

The GRF was significantly lower in DM1 and BMD during heel strike and higher during mid-stance compared to HC (*P* < 0.05) (Fig. 5A and C).

These differences became clearer with increasing disease severity in both groups (Fig. 5B and D). In the case of BMD Vignos 4 patients, their profile during the stance phase displays a nearly uniform and consistent pattern (Fig. 5D).

Trunk vertical acceleration in both patient groups showed lower amplitude than HC (*P* < 0.05). It is closer to zero during foot flat events and lower at toe-off compared to HC (*P* < 0.05) (Fig. 5A and C). This trend is more pronounced among the BMD groups and is more remarkable with greater disease severity (Fig. 5D). BMD Vignos 4 patients demonstrate nearly constant trunk acceleration throughout the gait cycle, resulting in minimal variation in trunk velocity (Fig. 5D).

### Joint kinematics

**Figure 6:**
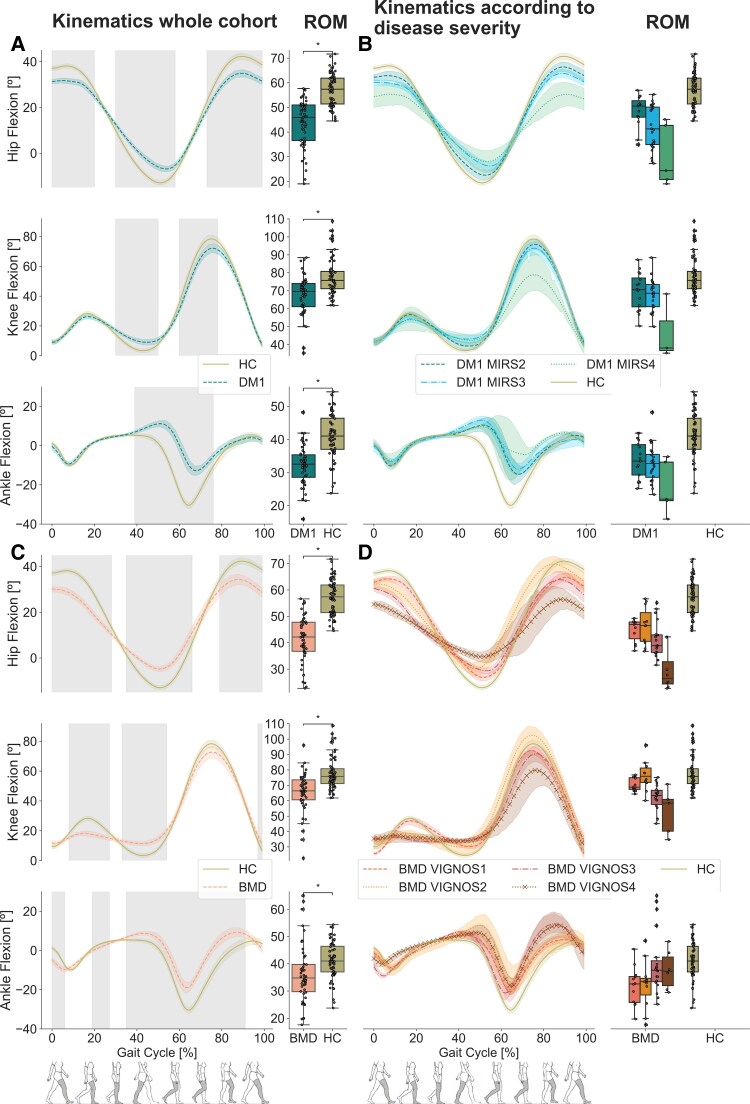
**Joint kinematics of the hip, knee and ankle.** (**A**) Joint kinematics (degrees of joint movement) in DM1 (blue striped line) for hip flexion, knee flexion and ankle flexion where the grey shaded area indicates a significant difference in comparison HC (olive solid line) during the walk test (*P* < 0.05) along with boxplots of range of motion for each joint in DM1 and HC. (**B**) Joint kinematics according to disease severity in DM1 (7 patients with severity 2, 11 patients with severity 3, 2 patients with severity 4) and HC along boxplots of range of motion for each joint in DM1 and HC. (**C**) Joint kinematics in BMD (orange striped line) for hip flexion, knee flexion and ankle flexion, where the grey shaded area indicates a significant difference in comparison with HC (olive solid line) during the test (*P* < 0.05) along with boxplots of range of motion for each joint in BMD and HC. (**D**) Joint kinematics in BMD according to disease severity (four patients with severity 1, four patients with severity 2, six patients with severity 3 and four patients with severity 4) and HC along with boxplots of range of motion for each joint in BMD and HC. Permutation and D Cohen effect size was used to analyse the grey shaded area, and Mann–Whitney U-test, corrected by multiple comparisons using the Bonferroni method, was used for the analysis for boxplots, comparing HC versus DM1 and HC versus BMD. Asterisk (*) indicates a significant difference (*P*_Bonferroni_ < 0.05). DM1, myotonic muscular dystrophy type 1; BMD, Becker muscular dystrophy; HC, healthy control; ROM, range of motion.

The DM1 and BMD patients presented significantly reduced range of motion (ROM) in all the joints evaluated: hip, knee and ankle (*P* < 0.05) (Fig. 6A and C).

In the DM1 group, joint kinematics exhibited significant differences when compared to HC and were characterized by reduced hip extension during pre-swing and initial swing and reduced hip flexion during swing, along with a restricted range of motion across the entire gait cycle. Furthermore, the DM1 group demonstrated decreased knee extension during terminal stance and reduced knee flexion during initial swing and diminished plantar flexion during pre-swing and initial swing (*P* < 0.05) (Fig. 6A). These differences increased progressively with disease severity. DM1 MIRS 4 also had higher knee flexion during mid-stance and less knee flexion in swing phase, reducing knee ROM, and higher dorsiflexion at mid-swing phase (Fig. 6B).

In individuals with BMD, joint kinematics differed significantly in comparison with HC. These differences were particularly prominent across the hip, characterized by reduced hip extension during pre-swing and initial swing and reduced hip flexion during swing, coupled with a diminished ROM throughout the entire gait cycle. Similarly, at the knee joint, the BMD group demonstrated less knee flexion during stance, and at the ankle joint, there was a notable decrease in plantar flexion during toe-off and initial swing (*P* < 0.05) (Fig. 6C). A visual interpretation showed that these differences increased progressively with disease severity, with BMD Vignos 4 showing much less hip extension compared to lesser affected patients. In the knee and ankle joints, there is a clear differentiation between BMD Vignos 1–2 from BMD Vignos 3–4, mainly presenting absence of knee flexion in the mid-stance phase and less knee flexion in swing phase and higher dorsiflexion at mid-swing phase (Fig. 6D).

## Discussion

Walking is one of the most essential motor functions of the human body. Therefore, assessing walking capability using the 6MWT is a desirable outcome to monitor disease course or treatment response in a variety of conditions affecting ambulation. The 6MWT performance is highly influenced by learning effects, patient motivation and day-to-day variation influenced by sleep, pain and mood, among many other factors. To mitigate day-to-day variation, a previous study introduced the use of HR correction to adjust for variability in exertion on separate days.^15^ Still, other factors affect walking capability, and especially in follow-up studies with disease deterioration or improvement by therapy, altered gait pattern or muscle activation may be key to understand disease progression or improvement, but such information is not always captured by a conventional 6MWT. Further, HR correction is not always possible in diseases with a high prevalence of cardiac involvement such as DM1 and BMD. Learning about biomechanics and biomechanical changes in gait patterns in individuals with muscle disorders is important because an increased knowledge about physical function and its progression can ultimately improve patient care. Walking difficulties can lead to mobility issues, balance deficits and increased risk of falls and potentially lead to a decline in independence and quality of life.

We conducted the current study to add more granularity to the interpretation of outcomes from the 6MWT by adding sensors to collect information about gait patterns, muscle activation and HR. We chose two muscle diseases with preferential distal (DM1) and proximal (BMD) involvement to capture objective findings in conditions with different walking patterns. The main findings of the study are that this non-invasive method, using data from movement and plantar pressure sensors, alongside sEMG findings and HR, contributes to a better understanding of the biomechanisms and muscle activation patterns that underlie the 6MWT. Our findings show that differences in walking distance are coupled with distinct patterns of walking between the two diseases. Importantly, different disease severities in both conditions can be observed using a qualitative analysis, which suggests responsiveness of the methodology in detecting real change in the clinical condition with progressive worsening or improvement by therapy.

For instance, a longer walking distance coupled with a higher HR but no change in the gait parameters assessed in this study is likely to be due to a higher exertion but no fundamental change in the condition. In contrast, a longer walked distance coupled with unchanged HR and gait parameters moving towards normalization likely reflects true improvement in the condition. The additional measures added to the 6MWT in this study will likely improve the interpretation of the results of the 6MWT in future trials. HR increased with higher disease severity in both patient groups, suggesting that severely affected patients worked at a much higher level of physical exertion even though absolute workload was lower. This attests to the usefulness of the HR to monitor changes in performance when this is unrelated to true changes in physical condition. However, as shown for the BMD patients with heart affection, the use of HR to help interpret results of the 6MWT is limited to patients with normal physiological responses to exercise, which is not the case when you are on beta-blockers, carry an ICD or pacemaker or have received a heart transplant. Cardiac arrhythmias could have been a confounding factor as well, especially in DM1 patients, but this was not encountered in the study. When cognizant of the limitations to the use of HR monitoring in people with heart involvement, HR is a strong endpoint to include in exertional tests of longer duration to correct/adjust for exertional strain during the test.

Patients suffering from muscle weakness present alterations in the gait pattern, due to compensatory strategies. One of these strategies is an increase in muscle co-activation, which may suggest that patients with muscular dystrophy, due to muscle weakness, require greater effort to start and complete a stride motion and stabilize joints. For the 6MWT, these alterations result in patients walking shorter distances than controls, and as disease severity increases, distance is further reduced. However, the 6MWT does not consider gait pattern characteristics.

Complementing the 6MWT with biomechanical sensors provides a deeper understanding of disease status and progression. The main compensatory strategies used by both the DM1 and BMD groups during walking have already been reported in the literature.^42-45^ In these studies, patients were tested in a biomechanical laboratory, where it is not possible to perform a 6MWT. A portable system, like the one used in this study, allows clinicians to evaluate patients in their facilities while performing clinical standard tests. Furthermore, the system can be utilized in different locations and spaces, giving clinicians and patients flexibility when performing functional assessments.

Myotonic dystrophy type 1 is characterized primarily by distal muscle affection and affection of bulbar muscles. This patient population exhibits not only muscle weakness but also myotonia, which may lead to tightness and rigidity, ultimately affecting locomotion due to affected limb range of motion. Clinically, gait pattern is characterized by short stride length and increased foot drop which can lead to increased ambulation difficulties and risk of falling.

Our findings show that during the toe-off/terminal stance and pre-swing phases, we observed a reduction of hip extension and higher amplitude in gluteus maximus, which suggests that due to muscle weakness and a potential tightness in the anterior part of the hip, the participants created a larger activation of their gluteus maximus to extend the hip resulting in a shorter stride length. Knee extension was similarly reduced, which suggests that the participants had increased tone in the hamstring muscles influencing the ability to sufficiently extend the knee during this phase of the gait cycle. We also observed a delayed and reduced plantar flexion, suggesting muscle weakness in anterior tibialis muscle, resulting in an altered movement pattern. Additionally, we observed a decreased ankle plantarflexion and dorsiflexion during toe-off and swing phase, suggesting the presence of myotonic muscles and foot drop. Despite our study protocol being different from previous studies, where we tested gait pattern during the 6MWT and Galli *et al*. and Wright *et al*. performed a self-selected pace walking test in a biomechanical laboratory, the similarities are noteworthy, such as limited ability to extend the hip and reduced hip motion, higher ankle dorsiflexion during stance and reduced plantar flexion at toe-off.^43,44^

The DM1 group exhibited a reduction in GRF during weight absorption (first peak) and an increase during mid-stance (valley) when the leg supports the entire weight. Additionally, a reduction in vertical acceleration is observed throughout the whole gait cycle. The decrease in force and deceleration in the first peak could be related to the reduction in walking speed and step length. Regarding the valley, the DM1 group showed weakness in the plantar flexor muscles, which in this phase are prime movers for controlling the movement of the tibia and facilitating the transfer of loads to the forefoot, ensuring knee extension. This weakness on the plantar flexions causes a slower transition from heel to toe and decrease in vertical downward acceleration, leading to a less extended hip and knee, and more dorsiflexed ankle position, which allows a higher force generation by the hip extensors. Finally, a larger stance phase was presented for the DM1 group. In DM1 group, we observed a similar activation of the proximal muscles as in HC despite the reduced hip and knee ROM, suggesting that this group needed higher activation to compensate for weak and myotonic muscles.

As disease severity increased on the MIRS scale in participants with DM1, the range of motion in the hip, knee and ankle progressively diminished, with greater differences observed between MIRS 2–3 and MIRS 4. Additionally, lower quadriceps activity and increased hamstrings activity during early swing were noted in patients with MIRS 3–4 but not in those with MIRS 2. This suggests that the participants compensated for a weak muscle by attempting to generate more power in the muscle during the gait cycle. However, it was difficult to interpret results from MIRS 4 patients because the group is represented by just two patients that were affected differently in the lower limbs, one with a severe distal weakness with gait limitations and the other patient without expressing that severity in the lower limbs. Both were classified as MIRS 4 since MIRS scale does not differentiate between upper and lower limbs weakness. These two patients showed different gait patterns in all the parameters evaluated, and there was a large difference in the distance walked, one was able to walk 600 m while the other around 200 m.

BMD is characterized by proximal muscle affection in the upper limbs and lower limbs. This disease along with DMD belongs to the same group of dystrophinopathies. Aside from proximal muscle affection, this patient group presents with a high prevalence of heart involvement. As the disease progresses, locomotion becomes altered and progressively difficult presenting as a waddling gait, where the patient exhibits a gait pattern which is characterized by walking on their toes and pushing their abdomen forward creating hyperlordosis in their lower back, to obtain balance and compensate for the lack of muscle strength.

In the BMD group, we observed a reduced hip motion throughout the gait cycle, indicating proximal muscle weakness and a diminished ability to generate locomotion. During the stance phase, we observed a straighter knee, which is consistent with the clinical description of the disease due to weakness in the anterior and posterior thigh muscles. This was further supported by sEMG amplitude, which depicts a lower amplitude in the quadriceps and hamstrings and a higher amplitude in the gluteus maximus, suggesting a potential compensation for these weak thigh muscles. Reduced range of motion in the hip and knee was also reported by O’Dowd *et al*.^45^ During terminal swing and ground contact, higher activation of hamstrings and gluteus maximus muscles was observed, suggesting leg deceleration and reduced weight absorption, as indicated by lower values in the GRF peak, similar to O’Dowd *et al*.^45^ During mid-stance, BMD group generated less downward acceleration, resulting in higher force values and earlier propulsion. This may cause BMD group to accelerate quicker during the propulsive phase to compensate for muscle weakness, requiring earlier preparation for forward motion. Overall, reduced trunk acceleration and GRF findings suggest a reduced ability to accept weight and absorb shock, leading to a reduction in gait velocity. As disease severity increased on the Vignos scale in participants with BMD, the participants exhibited an increasingly limited range of motion in the hip flexion suggesting muscle weakness during the forward motion of the leg. Participants with a Vignos 2 showed a reduction in knee flexion during stance phase, while participants with a Vignos 3–4 exhibited an extended knee which correlates well with the clinical description of the extended or hyperextended knee during the stance phase to compensate for the lack of muscle strength and obtain balance. In the initial contact phase, BMD group exhibited a flatter foot contact with the ground as severity increased. During foot clearance, participants with Vignos 3–4 compensated for their lack of proximal muscle strength by a pronounced ankle dorsiflexion. As disease progresses, differences observed in vertical trunk acceleration and GRF became more pronounced. The visual interpretations reported between BMD severity groups may suggest that some of these biomechanical parameters that change progressively are likely to be candidate outcomes to detect real changes over time or due to treatment.

In patients with DMD, it has previously been described how proximal muscles are hyperactivated to compensate for muscle weakness and how co-activating them ensures balance.^33^ In the BMD group, we observed a significantly greater co-activation of the hamstring and gluteus maximus muscles. Participants in this group with higher disease severity had a higher amplitude and earlier activation of quadriceps in the early swing phase and a decreased knee flexion during swing, which relates well to the findings reported in DMD.^33^ These findings confirm the presence of hyperactivation in these patients for compensating for muscle weakness.

Our study has some limitations. For instance, in the DM1 group, it would be interesting to investigate muscle activity of distal lower extremity muscles (gastrocnemius and tibialis anterior), as they are affected by the disease and involves toe-off and dorsiflexion of the foot for toe clearance.^46^ The participants in this study exhibited differences in cadence and stride length and these differences were significantly different from HC.^47-49^ A notable correlation has been identified between the cadence and stride length, with lower limb kinematics, kinetics and electromyography activities in both healthy and adults with different muscle conditions.^41-43^ With increasing stride length, knee flexion during stance, hip flexion during stance and hip extension during swing also increase, and peak ankle dorsiflexion at stance is reduced. Knee flexion and ankle dorsiflexion also increase when stride frequency increases. Muscle activity of the gluteus maximus, hamstring and quadriceps muscles have a higher activation amplitude when stride frequency or stride length is increased but the gait pattern is maintained.^46,47^ Peak vertical forces and vertical average loading rate are significantly higher when increasing walking speed, while stance duration is reduced.^48,49^ Differentiating between pathological differences or velocity differences can be challenging when the data contains differences in cadence and stride lengths. For example, in the BMD group, it was expected to have higher hip and knee flexion values during swing to compensate for foot drop; however, when comparing 6MWT gait patterns in BMD and HC groups, we observed that the HC group in fact used the same strategy to walk faster. But, since several parameters are recorded simultaneously, it is noticeable that peak hip flexion during late swing for Vignos 4 participants has a higher prominence, and the hip flexion occurs earlier to facilitate foot clearance.

Additionally, a study with a larger sample size is necessary to statistically confirm our visual findings. Future research could build on our findings by exploring how gait biomechanics change over the course of the 6MWT. Specifically, analysing the biomechanics throughout the entire test could provide valuable insights into gait variability, gait instabilities and the effect of fatigue on gait dynamics.

In this study, we used the Ephion Mobility system to establish a more objective and clinically relevant assessment of the 6MWT by adding measures of sEMG activity, joint kinematics and weight distribution during gait with this easy-to-use gait analysis system. Importantly, the system can provide a qualitative observation between disease severity groups, which suggests a responsiveness to change and thus usefulness as an important clinical endpoint in follow-up and therapeutic trials. Therefore, the system can be used to improve objectivity of the 6MWT regardless of factors that may influence the performance of the test, such as learning effect and patient motivation, and thus improve interpretability of the test. An improved objectivity of the 6MWT may also contribute to improving management of diseases in terms of the need for rehabilitation or assistive devices to improve patient care. Furthermore, the system can also be used in assessing ambulation with the use of other functional tests, for instance, the 2-min walk test or a self-paced walking test, in addition to a variety of other functions, such as rising from a chair and climbing stairs, and additionally be used in diverse patient populations.

## Data Availability

Data supporting the findings are available on reasonable request from the corresponding author.
